# MY LIFE PROFILE: EMPOWERING COMMUNITY-DWELLING OLDER ADULTS TO ADVOCATE FOR AGE-FRIENDLY HEALTH CARE

**DOI:** 10.1093/geroni/igae098.1519

**Published:** 2024-12-31

**Authors:** Gina Tucker-Roghi

**Affiliations:** Dominican University of California, San Rafael, California, United States

## Abstract

Older adults face a heightened risk of dementia and delirium, the latter affecting over half of hospitalized older adults, leading to longer hospital stays and poorer outcomes. Current evidence supports multi-component nonpharmacologic approaches such as cognitive activation, re-orientation, simple mobilization, social engagement and sleep hygiene to prevent delirium during hospitalization. Utilization of these non-pharmacological interventions may be enhanced by personal information. My Life Profile for Age-Friendly Care, rooted in the Age-Friendly Health Systems’ 4Ms framework and the Occupational Profile, serves as a proactive instrument. It is collaboratively developed by community-dwelling older adults and occupational therapy students, offering healthcare providers vital insights into the personal history, habits, routines, and preferences of older adults. Students are trained to utilize a semi-structured interview process to gather personal information relevant to Age-Friendly care. The student and older adult collaborate to compile the essential information onto a one-page profile, strategizing to ensure healthcare teams receive this information if hospitalization occurs. This process deepens students’ appreciation for personalized care and illustrates the significance of the Occupational Profile in driving person-centered care across healthcare teams. Feedback from 180 students over four years and participating older adults underscores the initiative’s effectiveness in affirming the value of personalized care in promoting older adults’ well-being and dignity. The My Life Profile for Age-Friendly Health Care project exemplifies how educational objectives and healthcare solutions can converge, highlighting the pivotal role of occupational therapy in fostering age-friendly healthcare practices.

